# Poly(ethylene glycol)-Prodrug Conjugates: Concept, Design, and Applications

**DOI:** 10.1155/2012/103973

**Published:** 2012-05-07

**Authors:** Shashwat S. Banerjee, Naval Aher, Rajesh Patil, Jayant Khandare

**Affiliations:** ^1^NCE-Polymer Chemistry Group, Piramal Life Sciences Ltd., 1 Nirlon Complex, Off Western Express Highway, Goregaon (E), Mumbai 400063, India; ^2^Semler Research Center Pvt Ltd., 75A, 15th Cross, I Phase, J. P. Nagar, Bangalore 560078, India

## Abstract

Poly(ethylene glycol) (PEG) is the most widely used polymer in delivering anticancer drugs clinically. PEGylation (i.e., the covalent attachment of PEG) of peptides proteins, drugs, and bioactives is known to enhance the aqueous solubility of hydrophobic drugs, prolong circulation time, minimize nonspecific uptake, and achieve specific tumor targetability through the enhanced permeability and retention effect. Numerous PEG-based therapeutics have been developed, and several have received market approval. A vast amount of clinical experience has been gained which has helped to design PEG prodrug conjugates with improved therapeutic efficacy and reduced systemic toxicity. However, more efforts in designing PEG-based prodrug conjugates are anticipated. In light of this, the current paper highlights the synthetic advances in PEG prodrug conjugation methodologies with varied bioactive components of clinical relevance. In addition, this paper discusses FDA-approved PEGylated delivery systems, their intended clinical applications, and formulations under clinical trials.

## 1. Introduction

The field of drug delivery system (DDS) utilizing polymeric carrier, which covalently conjugates molecule of interest, plays an important role in modern therapeutics [1, 2]. Such polymer-based drug entities are now termed as “polymer therapeutics” and include nanomedicine class that has become immensely critical in recent years [3–5]. The objectives for designing a polymer therapeutics are primarily to improve the potential of the respective drug by (i) enhancing water solubility, particularly relevant for some drugs with low aqueous solubility, (ii) stability against degrading enzymes or reduced uptake by reticulo-endothelial system (RES), and (iii) targeted delivery of drugs to specific sites of action in the body [1, 6].

**Figure 1:**
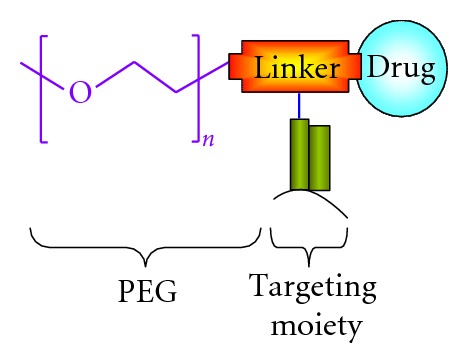
Schematic presentation PEG-based prodrug with targeting agent.

Poly(ethyleneglycol) (PEG) is the most commonly used nonionic polymer in the field of polymer-based drug delivery [1]. Due to high aqueous solubility, PEG polymer is considered as a versatile candidate for the prodrug conjugation. Ringdorf was the first to propose the rational model for pharmacologically active polymers in 1975 [7]. An ideal prodrug model typically consists of multiple components (Figure 1):

- polymer as a carrier;
- drug, peptide, or protein as a biological active component;
- spacer molecule or targeting moiety.

**Figure 2:**
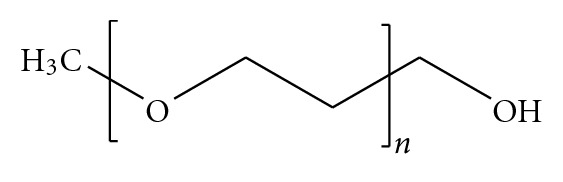
Molecular structure of monomethoxy PEG.

PEGylation, the covalent attachment of PEG to molecules of interest, has become a well-established prodrug delivery system [8, 9]. PEGylation was first reported by Davies and Abuchowski in the 1970s for albumin and catalase modification. Since then the procedure of PEGylation has been broadened and developed thereafter tremendously [10–16]. The remarkable properties of the biologically inert (biocompatible) PEG polymer derive from its hydrophilicity and flexibility. PEG is also considered to be somewhat hydrophobic due to its solubility in many organic solvents. Most used PEGs for prodrug modification are either monomethoxy PEG or dihydroxyl PEG (Figure 2) [7].

**Figure 3:**
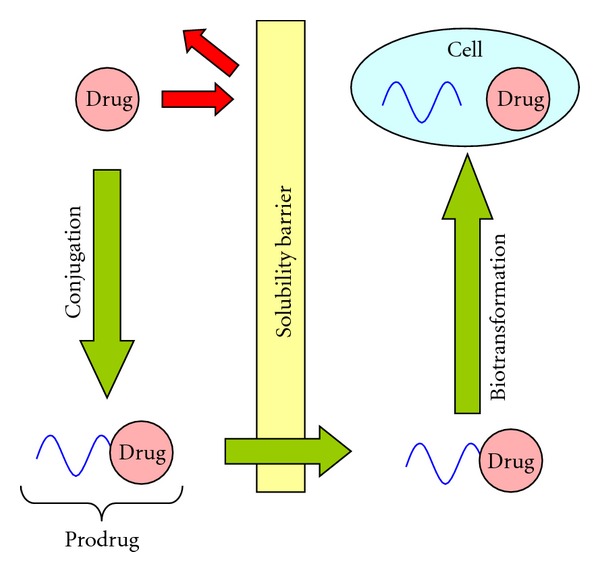
A schematic illustration of prodrug concept.

Typically, most of the PEG-based prodrugs have been developed for the delivery of anticancer agents such as paclitaxel, methotrexate, and cisplatin. High-molecular-weight prodrugs containing cytotoxic components have been developed to decrease peripheral side effects and to obtain a more specific administration of the drugs to the cancerous tissues [17]. Favorably, a macromolecular antitumor prodrug is expected to be stable in circulation and should degrade only after reaching the targeted cells or tissues. PEG-drug conjugates can therefore be tailored for activation by extra- or intracellular enzymes releasing the parent drug in situ (Figure 3) [7]. In this paper, we represent an overview on the advances of PEG prodrug conjugates which are being currently used as therapeutics. A short discussion with particular emphasis on the derivatives in clinical practice or still under clinical trials is also provided.

## 2. Properties of PEG

PEG in its most common form is a linear or branched polyether terminated with hydroxyl groups. PEG is synthesized by anionic polymerization of ethylene oxide initiated by nucleophilic attack of a hydroxide ion on the epoxide ring. Most useful for polypeptide modification is monomethoxy PEG (mPEG). On the other hand, mPEG is synthesized by anionic ring opening polymerization initiated with methoxide ions. Successful conjugation of PEG with biomolecule depends upon the chemical structure, molecular weight, steric hindrance, and the reactivity of the biomolecule as well as the polymer. In order to synthesize a bioconjugate, both chemical entities (i.e., the bioactive as well as the polymer) need to possess a reactive or functional group such as –COOH, –OH, –SH, or –NH_2_. Therefore, the synthetic methodology to form a conjugate involves either protection or deprotection of the groups [18].

## 3. PEG-Based Nanocarrier Architectures and Designs

There is need to design simple and yet appropriate PEG-conjugation methodology. Most commonly used strategies for conjugation involve use of both coupling agents such as dicyclohexyl carbodiimide (DCC) and 1-ethyl-3-(3-dimethylaminopropyl)carbodiimide (EDC) or use of *N*-hydroxysuccinimide (NHS) esters. Chemical conjugation of drugs or other biomolecules to polymers and its modifications can form stable bonds such as ester, amide, and disulphide. The resulting bond linkage should be relatively stable to prevent drug release during its transport until it reaches the target. Covalent bonds (e.g., ester or amide) are comparatively stable bonds and could deliver the drug at the targeted site. However, in some instances such bonds may not easily release targeting agents and peptides under the influence of acceptable environmental changes [19]. In the past, PEG prodrugs have been designed mostly for the delivery of anticancer agents due to its overall implications in the treatment. However it should be noted that PEG-antitumor prodrug is expected to be stable during circulation and degrade/hydrolyze only on reaching the targeted site. PEG-drug conjugates can therefore be tailored to release the parent drug *in situ* on activation by extra- or intracellular enzymes or pH change.

PEG has limited conjugation capacity since it possesses only one (two in case of modified PEGs) terminal functional group at the end of the polymer chain. To overcome this limitation of PEG, coupling amino acids, such as bicarboxylic amino acid and aspartic acid, to the PEG has been proposed [20, 21]. Such derivatization increases the number of active groups of the original PEG molecule. Using the same method with recursive derivatization, dendrimeric structures have also been achieved at each PEGs extremity. However, in the study the authors encountered low reactivity of the bicarboxylic acids groups towards arabinofuranosylcytosine (Ara-C) binding due to steric hindrance between two Ara-C molecules on conjugation with neighboring carboxylic moieties. It was suggested that this effect might be overcome by incorporating the dendrimer arms with an amino alcohol (H_2_N–[CH_2_–CH_2_–O]_2_–H).

**Figure 4:**
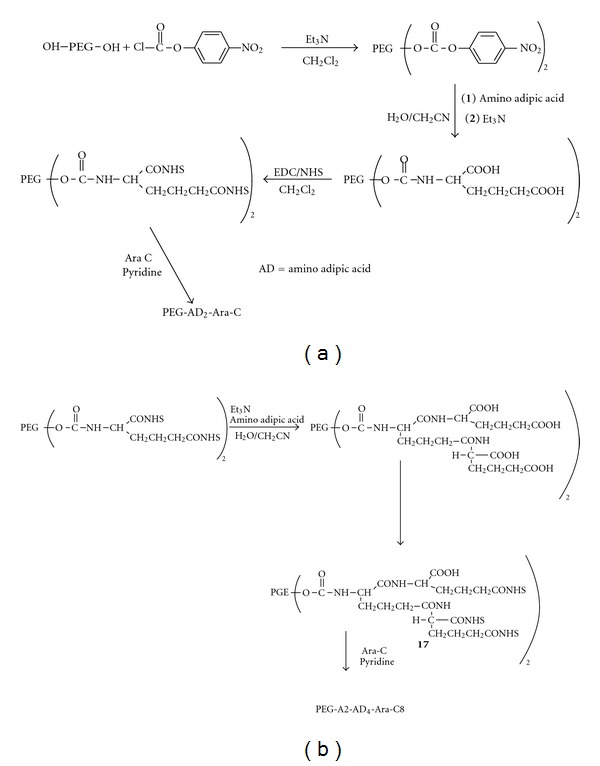
Synthetic schemes for PEG_10,000_-AD_2_-Ara-C_4_ (7) (a) and PEG_10,000_-AD_2_-AD_4_-Ara-C8 (8) conjugates (b). The antitumour agent 1-b-D-arabinofuranosylcytosine (Ara-C) was covalently linked to varying molecular weight –OH terminal PEGs through an amino acid spacer in order to improve the *in vivo* stability and blood residence time (reproduced from [22]).

PEG polymers with hydroxyl terminals can be easily modified by aliphatic chains molecules or small amino acids. For example, antitumor agent 1-*β*-D-Ara-C was covalently linked to varying molecular weight –OH terminal PEGs through an amino acid spacer in order to improve the *in vivo* stability and blood residence time [22]. Conjugation was carried out with one or two available hydroxyl groups at the polymer's terminals. Furthermore, to increase the drug loading of the polymer, the hydroxyl groups of PEG were functionalized with a bicarboxylic amino acid to form a tetrafunctional derivative. Finally, the conjugates with four or eight Ara-C molecules for each PEG chain were prepared (Figure 4). The authors investigated steric hindrance in PEG-Ara-C conjugates using molecular modeling to investigate the most suitable bicarboxylic amino acid with the least steric hindrance. Typically, hydroxyl groups of PEG are activated by *p*-nitrophenyl chloroformate to form a stable carbamate linkage between PEG and amino acid. The degree of PEG hydroxyl group activation with *p*-nitrophenyl chloroformate was determined by UV analysis of the *p*-nitrophenol released from PEG-*p*-nitrophenyl carbonate after alkaline hydrolysis. Activated PEG was further coupled with amino acid and the intermediate PEG-amino acid was linked to Ara-C by EDC/NHS activation.

### 3.1. PEG N-Hydroxysuccinimide (NHS) Esters and Coupling Methods

**Figure 5:**
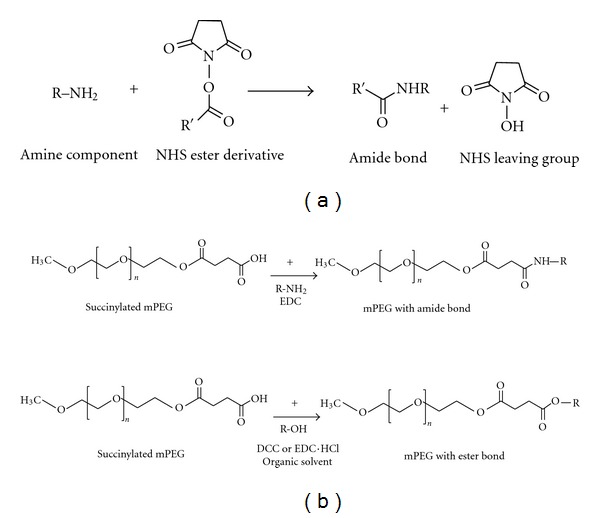
(a) NHS esters compounds react with nucleophiles to release the NHS leaving group and form an acetylated product. (b) PEG can be succinylated to form –COOH group, which can further form amide or ester bond with biomolecules.

**Figure 6:**
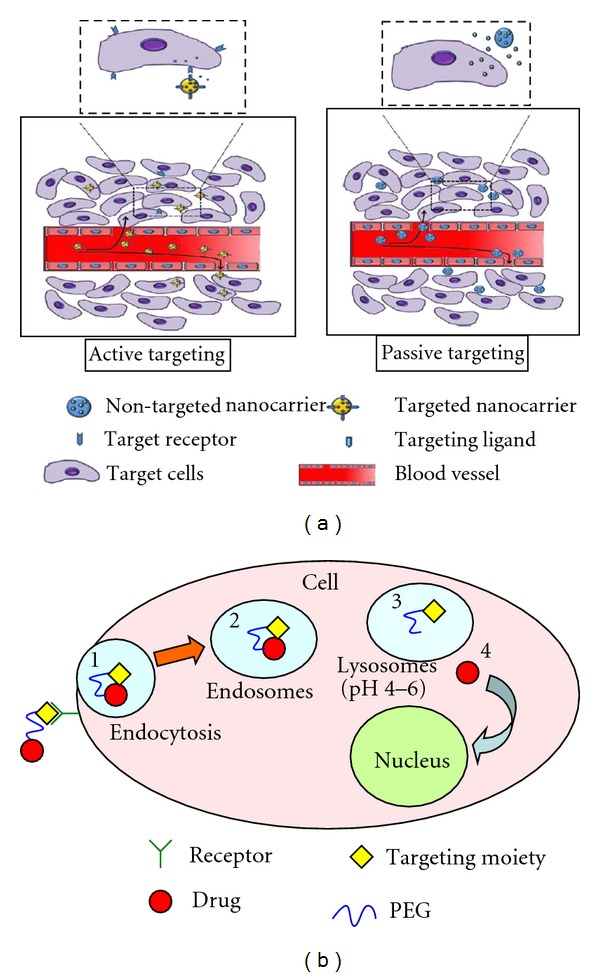
(a) Active and passive targeting by nanocarriers [35]; (b) (1) polymer-conjugated drug is internalized by tumor cells through receptor-mediated endocytosis following ligand-receptor docking, (2) transport of DDS in membrane limited organelles; (3) fusion with lysosomes; (4) the drug will usually be released intracellularly on exposure to lysosomal enzymes or lower pH (pH 6.5–<4.0) [31]. If the drug is bound to the polymer by an acid-sensitive linker then the extracellular release of drug takes place, especially if the drug is trapped by the tumor for longer period of time.

PEG-NHS esters are readily available which are reactive with nucleophiles to release the NHS leaving group and forms an acylated product [23] (Figure 5(a)). NHS is a choice for amine coupling because of its higher reactivity at physiological pH reactions in bioconjugation synthesis. In particular, carboxyl groups activated with NHS esters are highly reactive with amine nucleophiles and are very common entity in peptides and proteins. Polymers containing reactive hydroxyl groups (e.g., PEG) can be modified to obtain anhydride compounds. On the other hand, mPEG can be acetylated with anhydrides to form an ester terminating to free carboxylate groups (Figure 6).

The reactive PEG and its derivatives succinimidyl succinate and succinimidyl glutamate are used for conjugation with drugs or proteins. The coupling reactions involving amine groups are usually of two types: (a) acylation, (b) alkylation. These reactions are comparatively efficient to form a stable amide bond. In addition, carbodiimide coupling reactions or zero lengths crosslinkers are widely used for coupling or condensation reactions. Most of the coupling methodologies involve use of heterobifunctional reagent to couple via modified lysine residues on one protein to sulphydryl groups on the second protein [24], while modification of lysine residues involves the use of a heterobifunctional reagent comprising an NHS functional group, together with a maleimide or protected sulphydryl group. The linkage formed is either a disulphide bridge or as a thioether bond, depending if the introduced group is either a sulphydryl or maleimide, respectively. The thiol group on the second protein may be an endogenous free sulphydryl, or chemically introduced by modification of lysine residues.

## 4. PEG Prodrug Conjugates as Drug-Delivery Systems

In general, low-molecular-weight compounds diffuse into normal and tumor tissue through endothelia cell layer of blood capillaries [7]. Conjugation of low-molecular-weight drugs with high-molecular-weight polymeric carriers results in high-molecular weight prodrugs (Figure 1). However, such conjugation substantially alters the mechanism of cellular internalization and accumulation. High-molecular-weight drugs are internalized mainly by endocytosis, which is a much slower internalization process over to simple diffusion. Hence in case of endocytosis higher drug concentration outside the cell is required to produce the same cellular effect as corresponding low-molecular-weight drug [7]. Therefore, higher-molecular-weight prodrugs displays lower specific activity compared to its free form of drugs. For example, polymeric anticancer prodrugs are generally less toxic when compared with its free form, yet require substantially higher concentrations inside the tumor to be cytotoxic. Compensation for this decrease in drug efficacy can be achieved by targeting a polymeric drug to the specific organ, tissue, and/or cell [7].

Following two approaches is generally used to target polymeric anticancer drugs to the tumor or cancer cells [25, 26]:

1. passive targeting,
2. active targeting.

### 4.1. Passive Drug Targeting: The EPR Effect

Passive targeting is a drug delivery approach in which drugs are delivered to the targeted site by conjugating with polymer which releases the drug outside the targeted site due to altered environmental conditions (Figure 6(a)). Tumors and many inflamed areas of body have hyperpermeable vasculature and poor lymphatic drainage which passively provides increased retention of macromolecules into tumor and inflamed area of body [27–30]. This phenomenon is called enhanced permeability and retention (EPR) effect [27]. It constitutes one of the practical carrier-based anticancer drug delivery strategies. EPR effect is primarily utilized for passive targeting due to accumulation of prodrug into tumor or inflamed area. Low molecular drugs covalently coupled with high-molecular-weight carriers are inefficiently eliminated due to hampered lymphatic drainage and therefore accumulate in tumors. While EPR effect enhances the passive targeting ability due to higher accumulation rate of drug in tumor and subsequently due to accumulation, prodrug slowly releases drug molecules which provide high bioavailability and low systemic toxicity [30].

Passive accumulation of macromolecules such as PEG and other nanoparticles in solid tumors is a phenomenon which was probably overlooked for several years as a potential biological target for tumor-selective drug delivery. The existence of the EPR effect was experimentally confirmed by David et al., for macromolecular anticancer drug delivery systems [31]. Furthermore, passive targeting increases the concentration of the conjugate in the tumor environment and therefore “passively” forces the polymeric drug to enter the cells by means of the concentration gradient between the intracellular and extracellular spaces and therefore is not very efficient. The more efficient way to provide targeting is by “active targeting” [32].

### 4.2. Active Targeting

Active targeting approach is based on interaction between specific biological pairs (e.g., ligand receptor, antigen antibody, enzyme substrate) (Figure 6(a)) [33]. Active targeting is achieved by attaching targeting agents that bind to specific receptors on the cell surface—to the prodrug by a variety of conjugation chemistries. Most widely used targeting moieties are peptide ligands, sugar residues, antibodies, and aptamers specific to particular receptors, selectins, antigens, and mRNAs expressed in targeted cells or organs. The targeted anticancer LHRH-PEG-CPT conjugate is an example of such targeted anticancer drug delivery system [7]. In this system, LHRH peptide is used as a targeting moiety to the corresponding receptors overexpressed in several cancer cells, PEG polymer—as a carrier and CPT—as an anticancer drug. Interaction of these targeting moieties to their target molecule results in uptake of the drug by two main approaches: (i) internalization of the whole prodrug or (ii) internalization of the drug into targeted cells by various endocytosis and phagocytosis pathways [34].

**(i) Internalization of the Prodrug**

In this system, the drug is cleaved intracellularly after endocytosis. The internalized prodrug exhibits pharmacological activity on reaching the cytosol or the nucleus, which are the sites of action of intracellularly active drugs. This process can be divided into several distinct steps as schematically presented in Figure 6(b). Interaction of a targeted prodrug with a corresponding receptor initiates receptor-mediated endocytosis by formation of an endocytic vesicle and endosomes-membrane-limited transport vesicles with a polymeric delivery system inside [6]. The activity of the drug is preserved during the intracellular transport as the membrane-coated endosome prevents drugs from degradation by cellular detoxification enzymes. Endosomes fuses with lysosomes forming secondary lysosomes. If the drug-polymer conjugate is designed by incorporating an enzymatically cleavable bond then the drug is released from the polymer-drug conjugate by the lysosomal enzymes and might exit a lysosome by diffusion. The advantage of this approach is a high local drug concentration with a potential increase in efficacy [30].

**(ii) Internalization of the Drug**

In this system, the drug conjugate is cleaved extracellularly.

The microenvironment of tumors has been reported to be slightly acidic in animal models and human patients and the pH value in tumor tissue is often 0.5–1.0 units lower than in normal tissue.

## 5. Approaches and Applications

### 5.1. Polymer Conjugates of Therapeutically Relevant Proteins

The potential value of proteins such as antibodies, cytokines, growth factors, and enzymes as therapeutics has been recognized for years. However, successful development and application of therapeutic proteins are often impeded by several difficulties, for example, short circulating *t* *_1/2_,* low stability, costly production, poor bioavailability, and immunogenic and allergic potential. An elegant method to overcome most of these difficulties is the attachment of PEG chains onto the surface of the protein. PEGylation of the native protein generally masks the protein's surface, inhibits antibodies or antigen processing cells, and reduces degradation by proteolytic enzymes [6]. In addition, PEGylation of the native protein increases its molecular size and as a result prolongs the half-life *in vivo*, which in turn allows less frequent administration of the therapeutic protein.

**Figure 7:**
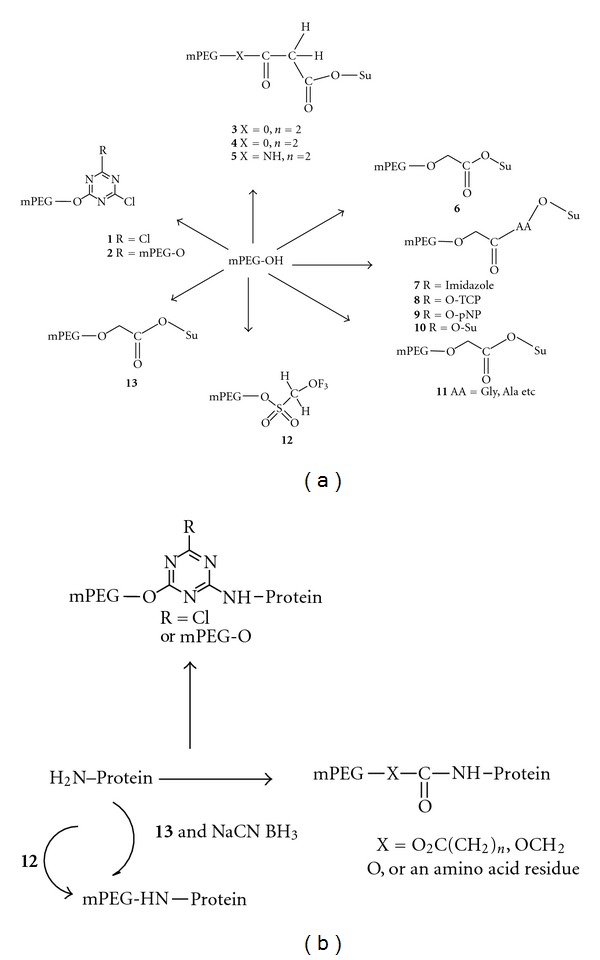
(a) mPEG-based protein-modifying methods. Protein modification with all of these agents results in acylated amine-containing linkages: amides, derived from active esters 3–6 and 11, or carbamates, derived from 7 to 10. Alkylating reagents 12 and 13 react with proteins forming secondary amine conjugation with amino-containing residues. As represented in (b) tresylate 12 alkylates directly, while acetaldehyde (13) is used in reductive alkylation reactions. The numbering (1–13) represent to the order in which these activated polymers were introduced (reproduced from [6, 36]).

The most common chemical approach for preparing PEG-protein conjugates has been by coupling –NH_2_ groups of proteins and mPEG with an electrophilic functional group [36]. Such conjugate reactions usually result in formation of polymer chains, covalently linked to a globular protein in the core. Figures 7(a) and 7(b) illustrate the commonly used methods of mPEG-based protein modifying reagents. Derivatives 1 and 2 contain a reactive aryl chloride residue, which is displaced by a nucleophilic amino group by a reaction with peptides or proteins, as shown in Figure 7(b). Derivatives 1 and 2 are acylating reagents, whereas derivatives 3–11 contain reactive acyl groups referenced as acylating agents. Protein modification with all of these agents results in acylated amine-containing linkages: amides derived from active esters 3–6 and 11 or carbamates derived from 7–10. Alkylating reagents 12 and 13 react with proteins forming secondary amine conjugation with amino-containing residues. As represented in Figure 7(a), tresylate 12 alkylates directly, while acetaldehyde 13 is used in reductive alkylation reactions. Numbers 1–13 represent the order in which these activated polymers were introduced [6, 36].

**Figure 8:**
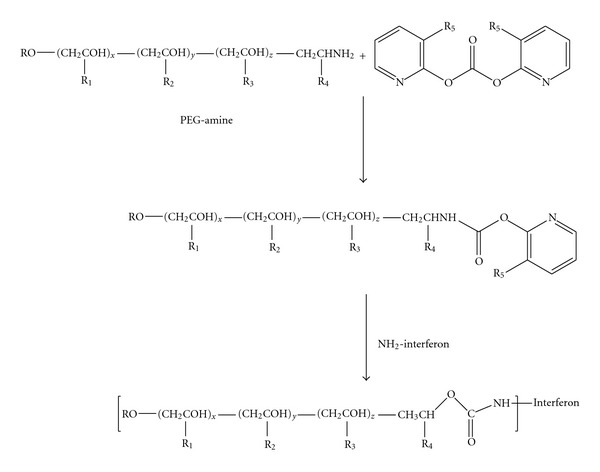
Synthesis of PEG-Intron by conjugating activated PEG with free amino groups in the interferon. R is lower alkyl group, R_1_, R_2_, R_3_, R_4_, R_1_′, R_2_′, R_3_′, R_4_′, R_5_ is H or lower alkyl; and *x*, *y*, and *z* are selected from any combination of numbers such that the polymer when conjugated to a protein allows the protein to retain at least a portion of the activity level of its biological activity when not conjugated; with the proviso that at least one of R_1_, R_2_, R_3_, and R_4_ is lower alkyl (reproduced from [40].

Adagen (pegademase bovine), used for the treatment of severe combined immunodeficiency disease (SCID), is developed using PEG polymer. PEG chemistry may results in side reaction or weak linkages upon conjugation with polypeptides and low-molecular-weight linear PEGs (≤12 kDa). It is prepared by first reacting mPEG (Mw 5000 Da) with succinic anhydride spacer. The resulting carboxylic group of PEG succinic acid is activated with *N*-hydroxysuccinimide (NHS) by using carbodiimide coupling agents. The NHS group is displaced by nonspecific reaction with nucleophilic amino acid side chains [37]. Another PEG prodrug of Enzon (Oncaspar^®^) is also synthesized by the use of PEG succinimidyl succinate [37]. The PEG ester and thioesters are highly susceptible to hydrolysis and thus modification occurs primarily at the amines forming amides. The PEGylated CERA protein conjugate, a product of Hoffmann-LaRoche (Mircera) is synthesized by attachment of an NHS-activated monomethoxy PEG butanoic acid to lysine 46 and 52 on erythropoietin (EPO) [38, 39]. Also, Hoffman-La Roche, Inc.'s peginterferon *α*2a (Pegasys) is prepared by conjugating PEG with the side chain and *N*-terminal amine groups of lysine spacer, forming a biscarbamate. Then on activation of the carboxylic acid with NHS, it helps the branched PEG chain linker form stable amide bonds with 11 possible lysine residues. Monosubstituted conjugate can also be synthesized by the same reaction process by limiting the amount of PEG chain linker used in the conjugation step. While, PEG-Intron by Schering-Plough (peginterferon *α*2b) is a covalent conjugate of interferon alfa-2b linked to a single unit of Mw 12000 PEG [40] is a covalent conjugate of interferon alfa-2b linked to a single unit of Mw 12000 PEG. The interferon conjugates are synthesized by condensing activated PEG, wherein a terminal hydroxy or amino group can be replaced by an activated linker, and reacting with one or more of the free amino groups in the interferon (Figure 8). Condensation with only one amino group to form a monoPEGylated conjugate is a prime feature of this synthesis process.

In other instance, pegvisomant (Somavert) prodrug conjugate is synthesized by covalent attachment of four to six Mw 5000 Da PEG units via NHS displacement to several lysine residues available on hGH antagonist B2036, as well as the *N*-terminal phenylalanine residue is used for acromegaly treatment [41–43]. Similarly, Amgen's pegfilgrastim (Neulasta^®^) is used to decrease febrile neutropenia manifested infection and this prodrug is a covalent conjugation of Mw 20000 Da monomethoxy PEG aldehyde by reductive amination with the *N*-terminal methionine residue of the filgrastim protein [44]. On the other hand, Krystexxa (pegloticase) by Savient, used for the treatment of chronic gout, is synthesized by using PEG *p*-nitrophenyl carbonate ester [45]. The primary amine lysine side chain is replaced by *p*-nitrophenol to form carbamates, which are further subjected to decrease hydrolysis under mild basic conditions. From the total of 28-29 lysines, approximately 12 lysines on each subunit of urate oxidase are surface accessible in the native tetrameric form of the complete enzyme. In fact, due to the close proximity of some of the lysine residues, PEGylation of one lysine may sterically hinder the addition of another PEG chain [45, 46].

### 5.2. PEG-Drug Conjugates

PEGylation of drugs does influence the pharmacokinetic properties of drugs and drug carriers and therefore is emerging as an important area in pharmaceutics. PEG has been successful for protein modification but in the case of low-molecular-weight drugs it presents a crucial limit, the low drug payload accompanying the available methoxy or diol forms of this polymer. This intrinsic limitation had for many years prevented the development of a small drug-PEG conjugate, and also because the conjugates extravasation into tumors by EPR effect is directly proportional to the conjugate's molecular weight. Unfortunately, in case of PEG the use of larger polymer does not correlate well with an increase in the amount of drug selectively delivered into the tumor. In case of PEG, the number of available groups for drug coupling does not change with the length of polymeric chain, as happens instead with other polymers (e.g., polyglutamic acid, and dextran) or copolymers (e.g., HPMA). The latter can have several functional groups along the polymeric backbone: longer polymer chains correspond to an increased number of functional groups [22, 47–49].

**Figure 9:**
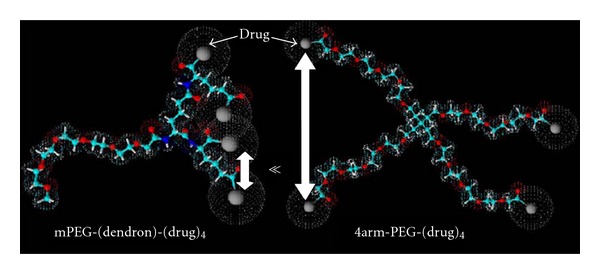
Schematic representation of higher steric entanglement in PEG dendrons with respect to multiarm PEGs (reproduced from [52]).

**Figure 10:**
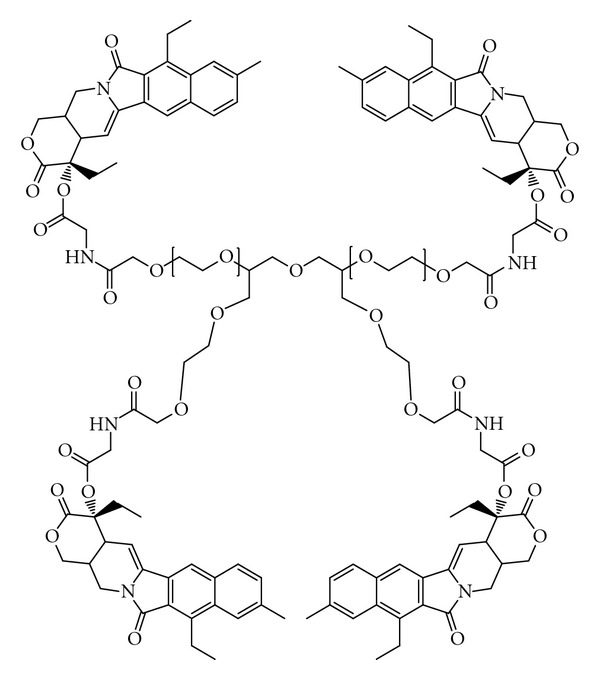
ENZ-2208: ^4°K^4 arm-PEG-(SN38)_4_ (reproduced from [53]).

A few studies have been conducted recently to overcome the low PEG loading by using multiarm PEGs either branched at the end chain groups or coupling on them small dendron structures (Figure 9) [47, 49–51]. Such multiarm PEG conjugates have recently entered phase I clinical trials [52]. This compound was obtained by coupling a 4-arm PEG of 40 kDa with the camptothecin derivative SN38, through a spacer glycine (Figure 10). The coupling strategy was developed to link selectively the 20-OH group of SN38, thus preserving the E ring of SN38 in the active lactone form while leaving the drug 10-OH-free [53].

Design and synthesis of nontargeted or antibody targeted biodegradable PEG multiblock coupled with *N*_2_,*N*_5_-diglutamyllysine tripeptide with doxorubicin (Dox) attached through acid-sensitive hydrazone bond has also been reported [54–57]. PEG activated with phosgene and NHS was reacted with –NH_2_ groups of triethyl ester of tripeptide *N*_2_,*N*_6_-diglutamyllysine to obtain a degradable multi-block polymer. The polymer was converted to the corresponding polyhydrazide by hydrazinolysis of the ethyl ester with hydrazine hydrate. On the other hand, the nontargeted conjugate was prepared by direct coupling of Dox with the hydrazide PEG multi-block polymer. Whereas the antibody-targeted conjugates, a part of the polymer-bound hydrazide group, was modified with succinimidyl 3-(2-pyridyldisulfanyl) propanoate to introduce a pyridyldisulfanyl group for subsequent conjugation with a modified antibody. Dox was coupled to the remaining hydrazide groups using acid-labile hydrazone bonds to obtain a polymer precursor. In addition, human immunoglobulin IgG modified with 2-iminothiolane was conjugated to the polymer by substitution of the 2-pyridylsulfanyl groups of the polymer with –SH groups of the antibody. It was demonstrated that Dox was rapidly released from the conjugates when incubated in phosphate buffer at lysosomal pH 5 and 7.4 (blood).

### 5.3. Incorporation of Spacers in Prodrug Conjugates

To construct a prodrug, various spacers have been incorporated along with the polymers and copolymers to decrease the crowding effect, to increase the reactivity, and reduce steric hindrance [6, 58]. The application of a spacer arm can enhance ligand-protein binding and also provide multiple binding sites. Ideal spacer molecules possess the following characteristics:

1. stable during conjugate transport,
2. adequate drug conjugation ability and,
3. being able to release the bioactive agent at an appropriate site of action.

Amino acid spacers such as alanine, glycine, and small peptides are most commonly used due to their chemical versatility for covalent conjugation and biodegradability. Heterobifunctional coupling agents containing succinimidyl have also been used frequently as spacers.

Polymer spacers are used to enhance the conjugation ratio of an antibody with a drug by introducing them between the targeting antibody and the drug. The use of an intermediate polymer with drug molecules carried in its side chains increases the potential number of drug molecules able to attach to that antibody by modification of only a minimum amount of existing amino acid residues (Figures 7(a) and 7(b)) [59].

## 6. PEG Therapeutics: Clinical Applications and Challenges for Development

PEG-based therapeutics were initially dismissed as interesting, but impractical to be translated in clinical setups. However, a growing number of products have shown that they can satisfy the stringent requirements of regulatory authority approvals (Table 1). Clinically used PEG conjugates are described below.

### 6.1. PEG-Proteins Conjugate

#### 6.1.1. Adagen (mPEG per Adenosine Deaminase)

Enzon's Adagen was among the first few PEG-protein conjugates to enter the clinic with FDA approval in 1990 [37]. It is used as a placement therapy to treat severe combined immunodeficiency (SCID) disease. SCID is an autosomal recessive genetic disorder caused by adenosine deaminase deficiency. It is usually fatal in children unless the patient is kept in protective isolation or undergoes a bone marrow transplant. As an alternative, Adagen is administered intramuscularly every 7 days. It is a replacement therapy and is repeated for the rest of the life by the patients following the dosing schedule: 10 U kg^−1^, 15 U kg^−1^, and 20 U kg^−1^ for the first three doses, and the weekly maintenance dose of 20 U kg^−1^. However, immune related problems have been reported for pegademase and its long-term treatment benefits are yet to be elucidated. Also, the high cost of treatment ($200,000–$300,000 per annum per patient) is an obvious disadvantage [60–62].

#### 6.1.2. Oncaspar^®^ (mPEG-*L*-Asparaginase)

Oncaspar (pegaspargase) is an antineoplastic drug from Enzon Pharmaceuticals Ltd. and was approved by FDA in 1994. Oncaspar is a PEG-modified entity of the enzyme *L*-asparaginase and is used for the treatment of acute lymphoblastic leukaemia [63]. PEGylation was attempted to overcome several factors limiting the utility of asparaginase as therapeutic agent such as high clearance, immunologic factors such as antibodies to asparaginase owing to bacterial protein and also inactivation due to conversion to asparagine via asparagine synthetase. Also, the immunological side effects such as hypersensitivity reactions (up to 73%) were major factors that limited clinical utility of *L*-asparaginase [64].

Pegaspargase was developed in the 1970–1980 while it was translated in the clinical trials in the 1980. Taking clues from the preclinical studies, a series of systematic clinical studies revealed the effectiveness of the pegaspargase as compared to its non-PEG-grafted parent drug [65, 66]. Clinical trials demonstrated safety in terms of fewer incidence of hypersensitivity reactions and prolonged duration of action. The trials defined different protocols (weekly or every two weeks) and recipes of multidrug regime to treat different malignancies. The clinical observations from clinical studies for pegaspargase conjugate are summarized in Table 2 [67, 68].

#### 6.1.3. Mircera (Continuous Erythropoiesis Receptor Activator or Methoxy Polyethylene Glycol-Epoetin Beta)

Mircera is a PEGylated continuous erythropoietin (EPO) receptor activator (CERA) introduced by Hoffmann-La Roche. It got approved by FDA in 2007 and is currently used to treat renal anemia in patients with chronic kidney disease (CKD). PEGylation of erythropoietin helps to prolong the half-life to approximately 130 h [69]. Darbepoetin alfa (Aranesp, Amgen), a second-generation EPO, due to the inclusion of an amino acid mutation has a higher glycosylation rate, and hence requires only weekly or biweekly injections. On the other hand, third-generation EPO (CERA) requires only monthly administration and thus helps in significantly improving the quality of life. However, it has been reported to have negligible effects on morbidity or mortality like other ESAs [70].

#### 6.1.4. Pegasys (Peginterferon Alfa-2a)

Pegasys (peginterferon alfa-2a) (Hoffmann-La Roche) drug is used to treat chronic hepatitis C (HCV) either alone or in combination with antimicrobial ribavirin. Pegasys was approved by FDA in 2002. It consists of a PEGylated interferon alfa-2a intended to mediate antiviral immune response. PEGylated interferon demonstrated higher efficacy by increasing the clearance time of the protein, thus maintaining interferon concentration levels in the blood to control HCV. The clinical study of peginterferon revealed that 180 *μ*g of peginterferon alfa-2a, administered once a week in patients with hepatitis C-related cirrhosis or bridging fibrosis was significantly more effective than 3 million units of standard interferon alfa-2a [71–73].

#### 6.1.5. PEG-Intron (Peginterferon Alfa-2b)

PEG-Intron [74] marketed by Schering-Plough is used to eradicate hepatic and extrahepatic hepatitis C virus infection. PEG conjugated with *α*-interferon (IFN) was approved by FDA for use in 2001. Monomethoxy-PEG-linked interferon has a sustained serum for 48–72 h compared to the native protein half-life of 7–9 h. The recommended dosage for standalone PEG-Intron therapy is 1 mg kg^−1^ per week for 52 weeks on the same day of the week subcutaneously [74, 75].

Interestingly, peginterferon *α*-2a has a higher market share because peginterferon **α**-2b is dosed on a body weight basis, whereas peginterferon *α*-2a is not. As a result, peginterferon **α**-2a is more frequently utilized to treat hepatitis C [68]. Nevertheless, some reports have suggested that peginterferon **α**-ribavirin combination therapy has higher risks of neutropenia and thrombocytopenia than interferon **α**-ribavirin combination therapy [87, 88], although both therapies have been reported to have similar side effect profiles.

#### 6.1.6. Somavert^®^ (Pegvisomant)

Pegvisomant (Somavert^®^) conjugate (Pfizer) is used to treat acromegaly by preventing human growth hormone (hGH) binding to its receptor, because this binding activates the signal pathways that lead to IGF-1 generation. It is a genetically engineered analogue of hGH conjugated with PEG which was approved for use in 2003 [89]. Acromegaly is a chronic metabolic disorder caused when the pituitary gland generates excess hGH after epiphyseal plate closure. GH receptor has two binding sites: (i) binds to site 1 and (ii) then to site 2, inducing the functional dimerization of the hGH receptor. Pegvisomant inhibits the dimerization of the hGH receptor due to its increased affinity for site 1 of the hGH receptor [89]. With eight amino acid mutations at the site, and by the substitution of position 120 glycine to arginine, inhibits hGH receptor dimerization. Overall, PEGylation reduces the activity of the GH receptor antagonist. However, the 4–6 PEG-5000 moieties added to pegvisomant prolongs its half-life and allow once-daily administration immunogenicity as the rate of clearance from the body are greatly reduced, making it an effective drug against acromegaly [90]. The recommended dosage for patients begins with subcutaneous administration of 40 mg dose. The patient can self-administer 10 mg of Somavert daily with adjustments to the dosage of Somavert in 5 mg increments depending on the elevation or decline of insulin growth factor-1 (IGF-I) levels [91, 92]. However, because pegvisomant can increase glucose tolerance, care is embarked for the diabetes mellitus patients [93].

#### 6.1.7. Neulasta (Pegfilgrastim)

Amgen's pegfilgrastim (Neulasta) is developed using filgrastim (Neupogen, Amgen) from Nektar (formerly Shearwater) PEGylation technology. The conjugate is formed by conjugating a 20 kDa linear monomethoxy-PEG aldehyde with Granulocyte-Colony Stimulating Factor G-CSF [94]. Neulasta is used to decrease febrile neutropenia manifested infection and was approved for use in 2002. The PEGylation increases the protein serum half-life to 42 h compared to the serum half-life of 3.5–3.8 h for the unmodified G-CSF. Therefore, the overall dose is reduced to a single cycle dose that is as effective as daily doses of native G-CSF [94–96]. The recommended dose of Neulasta is a single administration of 6 mg subcutaneously once-per-chemotherapy cycle and advised of not delivering it within 14 days before and 24 days after administration of chemotherapeutics [97].

#### 6.1.8. Krystexxa (Pegloticase)

Krystexxa (pegloticase) by Savient, a PEGylated mammalian urate oxidase (uricase) was FDA approved in 2010 [98]. It is a recombinant tetrameric urate oxidase used for the treatment of chronic gout. Pegloticase acts by preventing inflammation and pain due to urate crystal formation in plasma. The advantage of pegloticase over other standard treatments is the higher effectiveness in reducing gout tophi [99]. However, pegloticase has been reported to be immunogenic. Subcutaneous and intravenous injections of pegloticase in clinical trials showed production of antibodies [100–102]. However, it was found out that the antibodies produced were due to PEG and not because of uricase. Furthermore, as hydrogen peroxide may be produced during the conversion of uric acid to allantoin by uricase, the long-term safety profile of pegloticase needs to be established. Moreover, the transient local pain, slow absorption, and allergic reactions induced by subcutaneous injections of pegloticase were not observed after intravenous injections. However, intravenous injections are administratively inconvenient because self-administration is difficult and may have caused infusion reactions in multidose trials [103–105].

### 6.2. PEG-Drug Conjugates

PEG low-molecular-weight drug conjugates that entered the clinical trials are mostly from the camptothecin (CPT) family, namely, camptothecin itself, SN38, and irinotecan (Table 1). Although the first PEG based products were anticancer agents, subsequently other PEG therapeutics were developed and introduced for the treatment, for example, infectious diseases (e.g., PEG-interferons), and age-related diseases including macular degeneration and arthritis. Moreover, building of these first generation compounds, the pipeline of polymer therapeutics in clinical development continues to grow.

#### 6.2.1. Prothecan (PEG-Camptothecin)

**Figure 11:**
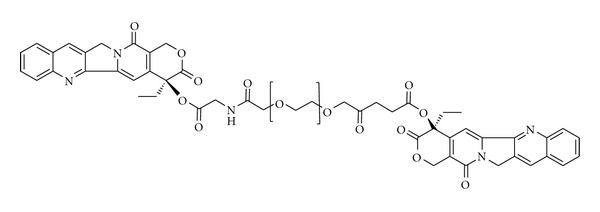
Synthetic structure of pegamotecan, a bisfunctional PEG-CPT conjugate mediated by a glycine spacer.

Pegamotecan is a product of Enzon Pharmaceuticals, Inc. which is PEG prodrug of the DNA damaging agent. The prodrug conjugate was conceived by coupling two molecules of CPT to a glycine-bifunctionalised 40 kDa PEG, yielding a drug loading of only approximately 1.7% (w/w) [105] (Figure 11). The CPT prodrug was designed with the aim of doubling the loading capacity to increase the drug half-life in blood by PEGylation and to stabilize CPT by acylation of the active lactone configuration of CPT [105]. The conjugation to PEG considerably enhanced CPT solubility and bioavailability at the tumor site. The maximum tolerated dose of the conjugate in phase I trials was determined at 7000 mg m^−2^ when administered for 1 h *i.v.* every 3 weeks, both for heavily and minimally pretreated patients. Phase I clinical studies underlined partial response in some cases and indicated that the conjugation to PEG notably improved the pharmacokinetics of the compound. Similarly, in phase II studies the same amount and administration schedule was recommended [106].

#### 6.2.2. NKTR-102 (PEG-Irinotecan)

The multiarm PEG design was employed for the synthesis of NKTR-102 by Nektar Therapeutics in which the drug was conjugated to a four-arm PEG for the treatment of solid tumors [107]. The plasma half-life evaluated for NKTR-102 in a mouse model taking into consideration the active metabolite SN-38, released from irinotecan demonstrated prolonged pharmacokinetic profile with a half-life of 15 days compared to 4 h with free irinotecan [53]. While in phase I clinical trial the safety, pharmacokinetic and antitumour activity of NKTR-102 were evaluated on patients with advanced solid tumors, (e.g., breast, ovarian, cervical, and non-small-cell lung cancer). Interestingly, 13 patients showed significant antitumor activity and reduction of tumor size ranging from a 40% to 58%, while 6 patients showed minor response only [22]. The cumulative SN38 exposure in patients treated with NKTR-102 was 1.2- to 6.5-fold higher than that predicted for irinotecan. The maximum tolerated dose (MTD) of the conjugate was to be 115 mg m^−2^ and the toxicity was manageable (diarrhea and not neutropenia is dose limiting). Noteworthy, that the patients enrolled in this study had failed the prior anticancer treatments or have tumors with no standard treatments available. Multiple phase II studies are ongoing with NKTR-102 alone or in combination with cetuximab for the treatment of ovarian, breast, colorectal, and cervical cancer [53].

#### 6.2.3. EZN-2208 (PEG-SN38)

The multiarm PEG-SN38 conjugate which recently entered phase I clinical trials (year) showed an increased drug loading of 3.7 wt.% with respect to pegamotecan. SN38 is an active metabolite of irinotecan and has 100- to 1000-fold more cytotoxic activity in tissue cell cultures than irinotecan. However, SN38 is practically insoluble in water and hence cannot be administered intravenously [53]. This PEG conjugation enhanced the solubility of SN38 by about 1000-fold. The conjugate acts as a prodrug system with a half-life of 12.3 min of SN38 release in human plasma. Even though the drug release is quite rapid, the PEG conjugate accumulates in tumor mass by EPR effect. In fact, EZN-2208 showed a 207-fold higher exposure to SN38 compared to irinotecan in treated mice, with a tumor to plasma drug concentration ratio increased over the time during the four-day-long pharmacokinetic and biodistribution studies [108]. Earlier, the derivatives demonstrated promising antitumor activity *in vitro* and *in vivo*. Especially, in mouse xenograft models of MX-1 breast, MiaPaCa-2 pancreatic, or HT-29 colon carcinoma, treatment with the conjugate administered either as a single dose or multiple injections exhibited better results than irinotecan [56]. However, recently Enzon Pharmaceuticals, Inc. announced the discontinuance of its EZN-2208 clinical program, following conclusion of its phase II study. The decision was taken in light of evolving standards of care for the treatment of metastatic colorectal cancer (mCRC). The company planned to continue to enroll studies for the other PEG-SN38 programs, which included a soon-to-be fully enrolled phase II study in metastatic breast cancer, a phase I study in pediatric cancer, and a phase I study in combination with Avastin (bevacizumab injection) in solid tumors [109].

## 7. Clinical Perspective

Early polymer therapeutics were developed as treatments for life-threatening diseases (cancer and infectious diseases), the emerging products, and clinical development candidates are designed for a much broader range of diseases. NKTR194, an opioid drug, being developed by Nektar using their advanced polymer conjugate technology platform is presently in the preclinical stage [110]. It has been designed to act peripherally without entering the CNS so that the gastrointestinal bleeding, CNS side effects, and cardiovascular risks associate with NSAIDs and COX-2 inhibitors used for treating moderate pains. NKTR-171 is another drug being designed by Nektar to treat neuropathic pain without CNS side effects is in the early research stage. NKTR-125 also in the research stage combines Nektar's PEGylation technology with potent antihistamine to enhance its anti-inflammatory properties and minimize the side effects.

BAX 855, Baxter's most advanced longer-acting candidate, is schedule to move into phase I clinical trial in 2011 [110]. It is a PEGylated FVIII molecule, which utilizes Nektar's PEGylation and Baxter's proprietary plasma and albumin-free platform. Preclinical animal studies have revealed that 1 injection of BAX 855 per week imparted similar FVIII levels as that of 3 injections of Advate given approximately every alternate day. In addition, Nektar and Baxter have collaborated to design long-acting clotting protein for hemophilia using Nektar's innovative PEGylation and releasable linker conjugate technology [110].

Convincingly, there are pioneering new approaches in research, for example, PEG-recombinant human HA-degrading enzyme, (rHuPH20) developed to degrade HA (it often accumulates in the tumor interstitium) with the aim of decreasing interstitial tumor pressure and to enhance penetration of both low-molecular-weight and nanosized anticancer agents [111, 112]. The latter provides an interesting opportunity for combination therapy.

## 8. Conclusions

PEG is currently the only water soluble polymer, widely accepted in therapeutics with market approval for different drugs. The reason for the wide utility of PEG is because its decreased interaction with blood components (low plasma protein binding) and high biocompatibility. PEGylated drugs such as peginterferon *α* and pegfilgrastim have proven their cost-effectiveness in the market, and products like pegvisomant and certolizumab pegol demonstrate that PEGylated forms will be marketed regardless of the prior commercialization of their non-PEGylated counterparts. This trend indicates that the long-term prospects for the biopharmaceutical PEGylated protein market are high. Due to significant clinical advantages, PEGylation is an essential proposition in delivering drugs and other bioactives. The therapeutic advantages of G-CSF, IFN, and EPO have been acknowledged, and PEGylation offers an attractive means of replacing the original market, given the assumption that biosimilars will appear soon after patents expire. Moreover, PEGylation allows drugs to be distinguished from simple biosimilars. The critical perspective of PEGylation is now envisioned to achieve cellular targetability and therefore suitable chemistry is being explored. Advanced forms of PEGs and their various architectures are designed and being introduced (e.g., hyper branched polyglycerols) [113]. Therefore, the importance of conducting comprehensive investigations on recently introduced potent peptides, proteins, oligonucleotides, and antibody fragments for PEGylation cannot be overemphasized.

## Figures and Tables

**Table 1:** PEG therapeutic systems with in the market or clinical development.

Product name	Description	Clinical use	Route of admin.	Stage
PEG-protein conjugates				

Oncaspar	PEG-asparaginase	Acute lymphocytic leukaemia	iv/im	Market
Adagen	PEG-adenosine deaminase	Severe combined immune deficiency syndrome	im	Market
Somavert	PEG-HGH antagonist	Acromegaly	sc	Market
PEGIntron	PEG-Interferon alpha 2b Hepatitis C	Hepatitis C	sc	Market
NeulastaTM	PEG-rhGCSF Chemotherapy	Chemotherapy-induced neutropenia	sc	Market
Pegasys	PEG-interferon alpha 2a hepatitis C	Hepatitis C	sc	Market
CimziaTM	PEG-anti-TNF Fab	Rheumatoid arthritis, Crohn's disease	sc	Market
Mircera	PEG-EPO	Anaemia associated with chronic kidney disease	iv/sc	Market
Puricase	PEG-uricase	Gout	iv	Market
Macugen	PEG-aptamer	Age-related macular degeneration	Intraviteal	Market

PEG-drug conjugates				

NKTR-102	PEG-irinotecan	Cancer-metastatic breast	iv	Phase II
PEG-SN38	Multiarm PEG-camptothecan derivative	Cancer-various	iv	Phase II
NKTR-118	PEG-naloxone	Opioid-induced constipation	Oral	Phase II

**Table 2:** Clinical trials and their outcome for pegaspargase conjugate.

Stage	Trial details	Observations/results	Reference
Phase I	31 patients with pegaspargase dose ranging from 500 to 8000 U m^−2^.	Mean half-life—357 h; dose unrelated hypersensitivity in small population of patients.	[67]
	Patients with advanced solid tumors; pegaspargase dose 250–2000 U m^−2^ every 14 days.	*L*-aspargine level were found to be very low which was again a function of dose. 2000 U m^−2^ dose showed adverse effects such as fatigue, nausea/vomiting and weight loss. Hence dose escalation beyond 2000 U m^−2^ was not evaluated.	[76]
	Low-dose (500 units m^−2^) in children with relapsed acute lymphoblastic leukemia.	*L-*asparaginase activity >100 U L^−1^ was demonstrated for atleast 1 week. Indicating in possibility reduction in dose.	[77]
	Five patients with AIDS related lymphoma treated with 1500 U m^−2^ every 2 weeks.	Three patients showed complete response.	[78]

	PEG-*L*-asparaginase as a single agent in patients (22) with recurrent and/or refractory multiple myeloma.	Maximal tolerated dose for single agent PEG-*L*-asparaginase in relapse/refractory multiple myeloma patients was found to be 1000 mg m^−2^ every 4 weeks.	[79]
Phase II	Patients earlier demonstrated sensitivity to *L-*asparaginase was treated with pegaspargase and other agents.	36% patients demonstrated complete response while 15% partial response.	[80]
	Newly diagnosed adults (14) with acute lymphoblastic leukemia (ALL) treated with 2000 U m^−2^ pegaspargase and multidrug regimen consisted of vincristine, prednisone, and danorubicin.	93% patients revealed complete response.	[81]
	Seven patients with refractory acute leukemias; dose 2000 U m^−2^ on days 1, 14, and 28 with other agents.	Five patients demonstrated complete response while one showed partial response.	[82]
	An open-label, multicenter study involving 21 patients with recurrent lymphoblastic leukemia with pegaspargase, 2000 U m^−2^ single dose. After 14 days patients were treated with multidrug therapy regime consisting of vincristine, prednisone, and some patients with doxorubicin and intrathecal therapy.	On day 14, 17% of patients (from 18) achieved complete response and 1% partial response. On day 35 (after the multidrug regime therapy), 67% patients demonstrated complete response and 11% showed partial response. The overall response rate was 78%.	[83]
	Pediatric oncology group study: patients with acute lymphoblastic leukemia treated with 2500 U m^−2^ with multidrug regime either weekly or every two weeks.	Highly significant 93% complete response was observed in the patients receiving weekly therapy as compared to 82% in patients receiving every two weeks.	[84]

Phase III	Reinduction of relapsed acute lymphoblastic leukemia: 2500 U m^−2^ pegaspargase on day 1 and 15 or 10,000 U m^−2^ *L*-asparaginase three times a week for 12 doses, both with multidrug regime.	Despite difference in dose and dosing rate the complete response and partial response rates were almost similar (63 and 65% for pegaspargase and *L-*asparaginase, resp.).	[85]
	Randomized trial involving Children with newly diagnosed acute lymphoblastic leukemia; 2500 U m^−2^ pegaspargase on day 1 or 6000 U m^−2^ *L*-asparaginase three times a week for three weeks.	Pegaspargase achieved faster rate of remission. Complete response rate was almost similar (98% versus 100% for pegaspargase and *L-*asparaginase, resp.) despite significant difference in dose and dosing rates.	[86]
